# Mitochondrial OPerA: tuning retrograde signaling and empowering intratumoral cDC1s

**DOI:** 10.1093/lifemeta/loag018

**Published:** 2026-06-22

**Authors:** Bhavana Kushwaha, Ruoning Wang

**Affiliations:** Center for the Childhood Cancer Research, Abigail Wexner Research Institute at Nationwide Children’s Hospital, Department of Pediatrics at The Ohio State University, Columbus, OH 43205, United States; Center for the Childhood Cancer Research, Abigail Wexner Research Institute at Nationwide Children’s Hospital, Department of Pediatrics at The Ohio State University, Columbus, OH 43205, United States

**Optic atrophy 1 (OPA1) is a dynamin-related guanosine triphosphatase (GTPase) located in the inner mitochondrial membrane that is critical for regulating mitochondrial fusion and cristae structure. You**  *et al.*  **reported that, in intratumoral conventional type 1 dendritic cells (cDC1s), OPA1 orchestrates mitochondrial retrograde signaling through nuclear respiratory factor 1 (NRF1) and ensures mitochondrial fitness and antitumor functionality. Notably, intratumoral administration of cDC1s with polarized mitochondria is sufficient to induce robust immunotherapeutic responses in preclinical settings, suggesting a novel “metabolic immunotherapy” strategy that may benefit cancer patients.** 

Tumors can co-opt multiple immunosuppressive mechanisms that act in concert to foster an immune-tolerant microenvironment and evade antitumor immune responses. Moreover, solid tumors impose a metabolically hostile environment on infiltrating immune cells. Cancer cells and immune cells share overlapping extracellular nutrient resources. As such, metabolically demanding cancer cells can restrict immune cell function by competing for nutrients and producing immunosuppressive metabolites [1]. Despite the clinical success of immunotherapies in some patients, more than three-quarters of cancer patients remain refractory to current immunotherapeutic regimens. Therefore, rational and effective metabolism-targeting approaches that enhance immune cell metabolic fitness and antitumor immunity may synergize with immunotherapy and improve clinical outcomes. Accordingly, various preclinical studies have demonstrated that modulating T-cell metabolism can significantly enhance antitumor immune responses, suggesting “metabolic immunotherapy” as a promi­sing strategy [2]. However, the application of these strategies to other intratumoral immune cell populations remains largely unexplored.

**Figure 1:**
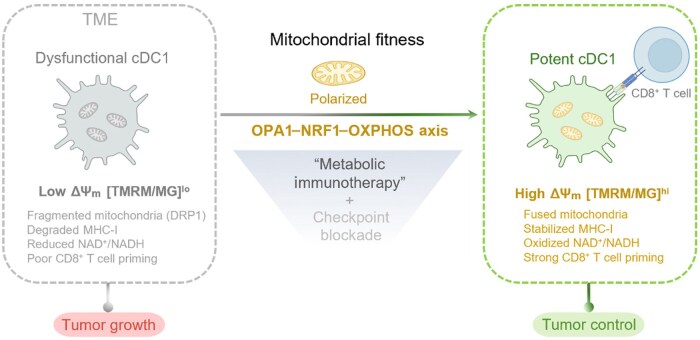
OPA1-driven mitochondrial fitness dictates cDC1 antitumor function. Dysfunctional [TMRM/MG]^lo^ cDC1s, characterized by a collapsed OPA1–NRF1–OXPHOS axis, exhibit enhanced MHC-I degradation, reduced NAD^+^/NADH ratios, and impaired CD8^+^ T-cell priming, thereby permitting tumor growth. In contrast, potent [TMRM/MG]^hi^ cDC1s maintain an intact OPA1–NRF1–OXPHOS axis, preserve antigen-presentation capacity and redox homeostasis, and effectively control tumor growth. Adoptive transfer of polarized [TMRM/MG]^hi^ cDC1s in combination with ICB (“metabolic immunotherapy”) converts the exhausted state into a potent antitumor state. Illustration created with BioRender.com.

Dendritic cells are critical for mounting adaptive immune responses and ensuring their exquisite specificity, magnitude, and quality. Metabolic rewiring, particularly of mitochondrial metabolism, in dendritic cells is required for mounting effective immune responses to infection [3]. Conventional type 1 dendritic cells (cDC1s) are essential for cancer immune surveillance and therapeutic responses. However, the solid tumor microenvironment (TME) renders cDC1s dysfunctional. Impaired cellular metabolism may contribute to the functional decline of cDC1s, thereby undermining responses to immunotherapy in cancer patients. In a recent study [4], You *et al.* reported that the gradual functional decline of intratumoral cDC1s is associated with reduced protein level of optic atrophy 1 (OPA1) and diminished mitochondrial fitness, as reflected by the reductions of mitochondrial membrane potential (ΔΨ_m_) and mitochondrial volume. Mechanistically, OPA1 orchestrates mitochondrial retrograde signaling through the transcription factor, nuclear respiratory factor 1 (NRF1), and endows intratumoral cDC1s with mitochondrial fitness and antitumor functionality. Therapeutically, intratumoral administration of cDC1s with polarized mitochondria, characterized by a high ratio of ΔΨ_m_ to mitochondrial mass (designated as [TMRM/MG]^hi^ cDC1s), is sufficient to induce robust immunotherapeutic responses, particularly in combination with immune checkpoint blockade (ICB), in preclinical settings, suggesting that enhancing cDC1 metabolic fitness may maximize the efficacy of cancer immunotherapies (Fig. 1).

To assess the metabolic status of intratumoral cDC1s, You *et al.* applied two widely used fluorescent dyes, tetramethylrhodamine methyl ester perchlorate (TMRM) and MitoTracker Green (MG), to concurrently measure ΔΨ_m_ and mitochondrial mass. This strategy uncovered a striking metabolic and functional dichotomy among intratumoral cDC1s. Metabolically fitter [TMRM/MG]^hi^ cells were markedly superior to their [TMRM/MG]^lo^ counterparts in priming CD8^+^ T-cell responses and restraining tumor growth. Unbiased proteomic profiling indicated that OPA1, a key regulator of mitochondrial fusion and cristae morphology, may determine the metabolic fitness of cDC1s. Accordingly, genetic deletion of *OPA1* impaired cDC1 antigen presentation, thereby dampening antitumor CD8^+^ T-cell immunity and accelerating tumor growth. Notably, OPA1 is also critical for the clonal expansion and survival of effector CD8^+^ T cells [5], suggesting that OPA1-dependent mitochondrial fitness is a shared feature of effector immune cell function. Collectively, these findings establish OPA1-governed mitochondrial fitness as a cell-intrinsic determinant of cDC1 immunogenicity and identify the [TMRM/MG]^hi^ state as a metabolically and functionally superior population that can be selectively exploited for therapeutic purposes.

Mechanistically, OPA1 deficiency compromised both the expression and transcriptional activity of NRF1, leading to defects in mitochondrial oxidative phosphorylation (OXPHOS), thereby establishing a retrograde signaling pathway that relayed inner mitochondrial membrane integrity to a nuclear transcriptional program. This finding extends the established role of OPA1 in shaping cristae architecture and respiratory chain efficiency to eliciting mitochondria-to-nucleus retrograde signaling through NRF1-dependent transcriptional control of the respiratory machinery [6]. Notably, the OPA1–NRF1 signaling axis, together with ΔΨ_m_ and mitochondrial volume, declined progressively during tumor progression, providing a mechanistic explanation for the gradual collapse of mitochondrial fitness and the functional decline of intratumoral cDC1s. By coupling mitochondrial status to a nuclear transcriptional program that governs bioenergetic output, this work defines a previously unrecognized OPA1–NRF1–OXPHOS signaling axis that dictates the metabolic and functional fitness of cDC1s, thereby representing a rational target for reversing intratumoral cDC1 dysfunction.

You *et al.* further delineated two effector arms through which OPA1-driven metabolism enforces cDC1 immunogenicity. First, the functional OPA1–NRF1 axis-mediated OXPHOS restrains the autophagy- and lysosome-dependent degradation of major histocompatibility complex class I (MHC-I) and captured antigens. Consequently, OPA1 loss unleashes autophagic-lysosomal turnover that depletes the MHC-I–peptide pool and dampens cross-presentation. Notably, the autophagy machinery is known to regulate surface MHC-I levels in both dendritic cells and tumor cells, where blocking such degradation restores surface MHC-I expression and synergizes with ICB to enhance CD8^+^ T-cell-mediated antitumor immunity [7–8]. Second, OPA1 is required to maintain the integrity of electron transport chain (ETC) complex I, thereby preserving NAD^+^/NADH balance. This finding indicates that the redox output of the respiratory chain, rather than ATP production alone, couples mitochondrial activity to cDC1 function. Supporting this notion, dietary NAD^+^ supplementation has been shown to synergize with immunotherapies in preclinical studies [9]. Collectively, these findings suggest that OPA1 simultaneously governs energy production, redox homeostasis, and the stability of the antigen-presentation machinery, making it a promising target for metabolically engineering cDC1s to enhance their functionality.

Finally, You *et al.* demonstrated that a better understanding of cDC1 metabolic fitness within the TME can enable the deve­lopment of rational and effective strategies to improve cancer immunotherapy. Intratumoral administration of cDC1s with polarized mitochondria, indicative of the [TMRM/MG]^hi^ state, is sufficient to induce robust immunotherapeutic responses. Moreover, this approach synergizes with ICB to elicit durable antitumor responses. The ΔΨ_m_-sensitive dye TMRM has also been used to isolate a metabolically distinct subset of CD8^+^ T cells. While one study showed that exhausted intratumoral CD8^+^ T cells are characterized by depolarized mitochondria, reflected by a low ratio of ΔΨ_m_ to mitochondrial mass [10], another study demonstrated that adoptive transfer of [TMRM]^lo^ T cells, but not [TMRM]^hi^ T cells, elicits robust antitumor responses associated with enhanced stem-like self-renewal capacity [11]. These findings suggest that mitochondrial polarization may reflect metabolic and mitochondrial fitness in an immune cell type-specific and context-dependent manner. Two recent studies found that impaired ETC activity caused by complex I or III deficiency dampens cDC1 metabolic fitness and antitumor functionality [12–13]. Further mechanistic analyses showed that a functional ETC establishes a poised DNA methylation landscape at specific genomic loci, thereby endowing cDC1s with the capacity for rapid activation. Together, these studies demonstrate that mitochondrial retrograde signaling serves as a critical surveillance mechanism that monitors mitochondrial metabolic status and fine-tunes the nuclear transcriptome to determine cDC1 antitumor responses. Collectively, these findings provide a rationale for targeting metabolism and mitochondrial retrograde signaling to reverse intratumoral cDC1 dysfunction, presenting a promising “metabolic immunotherapy” strategy to improve therapeutic outcomes in cancer patients.
